# Marine Polyphenols in Cardiovascular Health: Unraveling Structure–Activity Relationships, Mechanisms, and Therapeutic Implications

**DOI:** 10.3390/ijms25158419

**Published:** 2024-08-01

**Authors:** D. P. Nagahawatta, N. M. Liyanage, Thilina U. Jayawardena, You-Jin Jeon

**Affiliations:** 1 Department of Marine Life Sciences, Jeju National University, Jeju 63243, Republic of Korea; pramuditha1992@jejunu.ac.kr (D.P.N.); liyanagenm@jejunu.ac.kr (N.M.L.); 2 Department of Medicine, University of Alberta, Edmonton, AB T6G 2S2, Canada; 3 Marine Science Institute, Jeju National University, Jeju 63333, Republic of Korea

**Keywords:** marine polyphenolic compounds, cardiovascular diseases, structure–activity relationship

## Abstract

Cardiovascular diseases (CVDs) are responsible for significant mortality rates globally that have been raised due to the limitation of the available treatments and prevalence of CVDs. The innovative research and identification of potential preventives for CVDs are essential to alleviate global deaths and complications. The marine environment is a rich source of bioactive substances and provides a unique chemical arsenal against numerous ailments due to its unrivaled biodiversity. Marine polyphenolic compounds (MPCs) are unique because of their structural variety and biologically significant activity. Further, MPCs are well-reported for their valuable biological activities, such as anti-inflammatory, cardioprotective, and antioxidant, demonstrating encouraging results in preventing and treating CVDs. Therefore, investigation of the structure–activity relationship (SAR) between MPCs and CVDs provides insights that reveal how the structural components of these compounds affect their effectiveness. Further, comprehending this correlation is essential for advancing medications and nutraceuticals sourced from marine sources, which could transform the strategy for treating and preventing cardiovascular diseases. Therefore, this study provides a comprehensive analysis of existing research by emphasizing the role of MPCs in CVD treatments and evaluating the SAR between MPCs and CVDs with challenges and future directions.

## 1. Introduction

Cardiovascular diseases (CVDs) are a vital issue in the current world due to their significant mortality rate of around 17.9 million per year. The mortality projections exhibit that the number of deaths is currently increasing and will affect 23.6 million people by 2030 [1]. Beyond this statistic, several factors influence this increasing prevalence of cardiovascular diseases (CVDs), such as the growth and aging of the population. As the world’s population expands and ages, the number of individuals affected by CVDs naturally increases [2]. Further, there have been improvements in health status and quality of life in many developed countries. Advances in medical care, lifestyle changes, and public health initiatives have contributed to longer lifespans [3]. However, it is crucial to recognize that over three-quarters of CVD deaths occur in low- and middle-income countries [4]. Further, generation replacements and adaptation are also crucial for the above-mentioned statistical figures [5]. However, factors such as aging and generation continuity are unavoidable natural processes, and it is essential for managing age-related and non-related diseases including CVDs. This alarming trend underscores the urgent need for innovative prevention, treatment, and management approaches. The investigation of novel therapeutic agents has gained the attention of researchers and led them to extensively explore unknown marine environments, a reservoir of unique bioactive compounds with potential health benefits.

Marine ecosystems, renowned for their extraordinary biodiversity, are a rich source of numerous valuable bioactive compounds with complex structures that serve various pharmacological activities [6,7,8,9]. Among these, marine polyphenolic compounds (MPCs) have emerged as potent bioactive compounds and play a vital role in combating CVDs [10]. These compounds, derived from various marine sources, including seaweed and other marine plants, have been part of the human diet in coastal cultures for centuries [6,9]. MPCs, with their high structural diversity and the presence of multiple phenolic groups, are responsible for their biological activities [11]. The unique properties of marine polyphenols, stemming from the extreme conditions under which they are produced and their unique chemical structure, set them apart from their terrestrial counterparts. The multifaceted bioactivities of MPCs, including antioxidant, anti-inflammatory, antithrombotic, and lipid-modulating effects, all hold promise in the fight against CVDs [12,13,14,15].

Studying the structure–activity relationship (SAR) between MPCs and CVDs is a pivotal endeavor in the battle against cardiovascular diseases. Unraveling the intricate connection between the molecular structure and their biological effect is a solid pathway for developing novel and effective cardiovascular therapeutics. The high complexity of polyphenolic structures in the marine environment offers a unique and rich source for explorations. Understanding the SAR can lead to the identification of key functional groups and molecular configurations of MPCs that can be most effective in combating CVDs. This knowledge not only aids in the design of potent cardiovascular drugs but also contributes to the broader understanding of the disease mechanisms at a molecular level. Therefore, the continued investigation into the SAR of MPCs holds the promise of significant advancements in cardiovascular medicine, offering significant aid for better prevention, treatment, and management strategies for those affected by these pervasive diseases. The current study, as a crucial part of ongoing research, delves into the therapeutic potential of MPCs in the treatments for CVDs, revealing its SAR and paving the way for identifying current limitations and future potentials.

## 2. Marine Polyphenolic Compounds: Diversity and Sources

MPCs are secondary metabolites mainly found in algae that accumulate for defense and survival [16]. They exhibit various biological activities, including antioxidant, anti-inflammatory, and potential antiviral activity [12,13,14,15,17,18]. Due to their significant biological activities, marine polyphenols hold interest in potential pharmaceutical, nutraceutical, and cosmetic applications. They are being studied for their ability to protect against oxidative stress, inflammation, and microbial infections and their potential in skin care as protective agents against aging and wrinkles [19]. Studies suggest that MPCs can modulate blood pressure, improve lipid profiles, and stabilize atherosclerotic plaques, thereby reducing the risk factors associated with CVDs [10]. Their role in cardiovascular health is a growing area of interest, with ongoing research aimed at understanding the mechanisms behind their beneficial effects and developing them into therapeutic agents.

### 2.1. Chemical Classification

**Figure 1:**
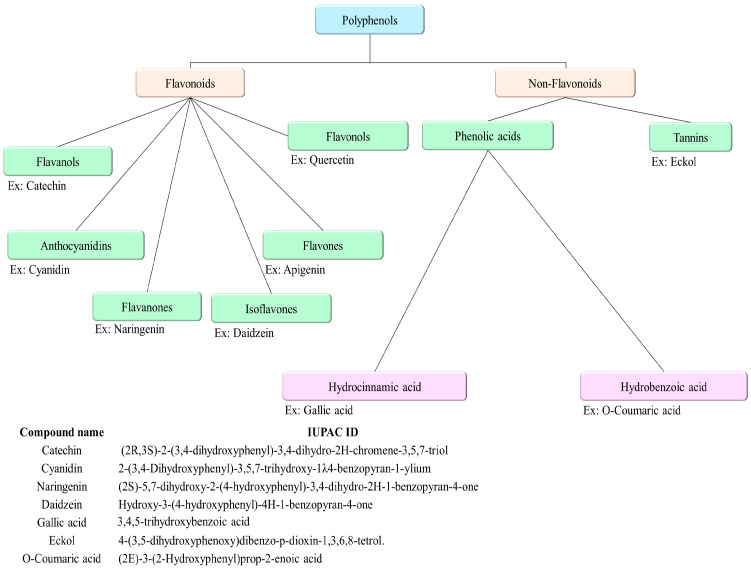
Chemical classification of polyphenols.

MPCs’ vast structures and biological activities are classified based on the number of phenol units within their molecular structure, substituent groups, and the linkage type between phenol units [20]. This classification is essential for understanding their potential as therapeutic agents, particularly in the context of CVDs. The classification is summarized in Figure 1 [21]. Polyphenols are mainly divided into flavonoids and non-flavonoids. However, some scientists have classified polyphenols into three categories: flavonoids, non-flavonoids, and phenolic acids. This classification is essential for understanding their potential as therapeutic agents, particularly in the context of CVDs.

The number of multiple phenol units can be varied, leading to a diverse range of structures with different biological activities. The type and position of substituent groups, such as hydroxyl, methoxyl, or glycosyl groups, significantly influence the properties of polyphenols. These groups can affect the compound’s solubility, stability, and ability to interact with biological targets [22]. The linkages between phenol units, such as ether or ester bonds, determine the compound’s structure and potential to form larger polymers. For example, tannins are known for their ability to form complex structures through inter-unit linkages. Moreover, polyphenolic compounds can range from simple monomers to complex polymers. The degree of polymerization impacts their bioavailability and biological effects. Higher polymerization often correlates with stronger antioxidant activities but may reduce bioavailability [22]. The biosynthetic pathways that produce these compounds also affect their classification. Polyphenols can be derived from different metabolic routes, such as the shikimate or polyketide pathways, leading to distinct structural families [23].

#### 2.1.1. Flavonoids

Marine flavonoids are a class of polyphenolic compounds that are synthesized by various marine organisms, including algae, seagrasses, and mangroves. These compounds are structurally similar to their terrestrial counterparts but are adapted to the unique conditions of the marine environment [24]. The chemical characteristics and properties of flavonoids depend on various factors.

The basic structure of marine flavonoids possesses the typical flavonoid structure of two phenyl rings (A and B) connected by a three-carbon chain that forms a heterocyclic ring, often denoted as C6-C3-C61. The A ring is usually a benzoyl moiety, while the B ring is typically a cinnamoyl moiety, and variations in these rings contribute to the diversity of flavonoids. The position and degree of hydroxylation on the rings, especially the B ring, are critical for the antioxidant activity of flavonoids. The more hydroxyl groups present, the higher the potential for free radical scavenging. Many marine flavonoids are glycosylated, meaning they have sugar molecules attached. This modification can affect their solubility, stability, and bioavailability. Methylation of hydroxyl groups can also occur, enhancing the lipophilicity of flavonoids and affecting their interaction with biological membranes. Further, some marine flavonoids have unique substitution patterns not commonly found in terrestrial plants, such as the presence of sulfate, chlorine, and amino groups. These modifications can significantly alter their biological activities. The solubility of flavonoids is influenced by their degree of glycosylation and methylation. Glycosylated flavonoids are more water-soluble, while methylated flavonoids are more lipophilic. Marine flavonoids can be sensitive to environmental factors such as light, temperature, and pH. Glycosylation can enhance their stability against oxidative degradation [24].

The antioxidant capacity of flavonoids is mainly dependent on their ability to donate hydrogen atoms or electrons to neutralize free radicals. The arrangement of hydroxyl groups, especially the ortho-dihydroxy configuration in the B ring, is crucial for this activity. Moreover, flavonoids can interact with various biological targets, including enzymes, receptors, and nucleic acids. These interactions are critical to their diverse biological effects, such as anti-inflammatory, anticancer, and cardioprotective actions [24]. The bioavailability of marine flavonoids is a complex issue, influenced by their chemical structure, metabolism, and interaction with gut microbiota. Glycosylation can enhance their absorption in the human gut.

Flavonoids are divided into six major groups [24]. Each group has specific chemical characteristics, and Table 1 summarizes the differences among these groups [24,25,26].

#### 2.1.2. Non-Flavanoids

Exploring the vast chemical diversity of MPCs reveals that the non-flavonoid constituents represent a remarkable and less explored domain of MNPs. These compounds, which include phlorotannin, marine tannins, and sulfated polyphenols, among others, are not only structurally distinct from flavonoids but also exhibit unique bioactivities that are pivotal to both the ecological roles they play in marine environments and their potential therapeutic applications [27]. Investigating the intricate world of marine non-flavonoid polyphenols, including their chemical characteristics and biosynthesis, provides promising avenues for pharmacological innovations.

Non-flavonoid polyphenols in marine sources, such as phenolic acids, stilbenes, and lignans, are renowned for their robust antioxidant activity. This activity, attributed to their phenol moiety and a resonance-stabilized structure that enables them to donate protons, is crucial in scavenging free radicals and protecting against oxidative stress [27]. These characters gained the attention of scientists because of their potential health benefits, underscoring the significance of our research on these compounds.

Phenolic acids are simple phenolic compounds that often act as precursors for more complex polyphenols. They typically contain one carboxylic acid group attached to an aromatic ring with one or more hydroxyl groups. Examples include hydroxybenzoic and hydroxycinnamic acids, known for their antioxidant properties. They possess a C6-C1 structure and are known for their antioxidant properties through radical scavenging mechanisms. Tannins are high-molecular-weight polyphenolic compounds that can be further divided into hydrolyzable and condensed tannins. They can bind and precipitate proteins and are known for their astringent properties [28].

Stilbenes can be characterized by a 1,2-diphenylethylene nucleus. Stilbenes, like resveratrol, are produced by plants in response to stress and have been studied for their anti-inflammatory and cardioprotective effects. These compounds have a C6-C2-C6 structure and are less common than other polyphenolic compounds. Further, stilbenes are known for their potential health benefits, including anti-inflammatory and anticancer activities [29,30].

Lignans are formed by the dimerization of two phenylpropanoid units. With a C6-C3-C6 structure, lignans are formed by the dimerization of two phenylpropanoid units. Secoisolariciresinol and matairesinol are lignans that offer health benefits such as anticancer, anti-inflammatory, and antioxidant activities [31,32].

Overall, marine polyphenols, including non-flavonoids, have unique structures that contribute to their biological activities. The structure of marine polyphenols can be complex, often containing multiple hydroxyl groups, which are responsible for their potent antioxidant properties. The structural complexity and diversity are due to the various environmental conditions marine organisms live in, leading to the production of unique polyphenolic compounds [23,33]. For instance, marine algae produce phlorotannins, a type of non-flavonoid polyphenol. These are unique to marine sources and are synthesized through the acetate–malonate pathway, also known as the polyketide pathway. The polymerization of phloroglucinol units forms phlorotannins and has been observed to possess anti-inflammatory and antioxidative properties. The comparative overview of non-flavonoids is summarized in Table 2.

### 2.2. Extraction, Isolation, and Purification of Marine Polyphenolic Compounds

Exploring marine polyphenolic compounds has become a frontier in searching for novel bioactive substances with potential therapeutic and industrial applications. The initial steps in utilizing these compounds involve a crucial process consisting of their extraction, isolation, and purification from marine matrices. Therefore, identifying suitable methodologies and technologies is essential to efficiently and sustainably extract these valuable compounds and isolate and purify them precisely to the highest standards, ensuring their optimal bioactivity and integrity for further use. The advancements in this field increase marine-derived innovations and underscore the importance of preserving the balance of marine ecosystems from which these potent compounds originate.

Extracting MPCs is a vital process that involves several steps to ensure the effective recovery of these valuable bioactive substances from marine sources [34]. The choice of solvent is critical in the extraction process. Solvents such as ethanol, methanol, acetone, and water are commonly used due to their ability to dissolve a wide range of polyphenolic compounds. The selection of solvent is based on the polarity of the target compounds, with more polar solvents being effective for more polar polyphenols [35].

Extraction techniques can be mainly divided into two categories: conventional and advanced methods. Conventional extraction methods include maceration, percolation, and Soxhlet extraction. While effective, they often require longer extraction times and larger volumes of solvents [36]. Advanced extraction methods, including techniques such as ultrasound-assisted extraction (UAE), microwave-assisted extraction (MAE), and supercritical fluid extraction (SFE), have been developed to improve efficiency. UAE uses ultrasonic waves to disrupt cell walls, enhancing solvent penetration [37]. MAE utilizes microwave energy to heat the solvent and plant matrix, speeding up the extraction process [38]. SFE, often using supercritical CO_2_, is highly selective and environmentally friendly [39]. Optimization of extraction conditions such as temperature, time, solvent-to-solid ratio, and particle size of the marine material is vital to maximize the yield [36]. Higher temperatures can increase polyphenols’ solubility and diffusion rate, but they must be carefully controlled to prevent the degradation of heat-sensitive compounds. The solvent-to-solid ratio ensures enough solvent to dissolve the polyphenols, while the particle size affects the surface area for extraction. Moreover, the efficiency of the extraction process is evaluated based on the yield of polyphenolic compounds recovered [36]. Advanced techniques like UAE and MAE provide higher yields in shorter times than conventional methods. The use of enzymes to break down cell walls and release bound polyphenols is also being explored to increase yields. After extraction, the solvent is typically removed by evaporation under reduced pressure, and the crude extract may undergo further purification steps to isolate specific polyphenolic compounds [40].

Isolation is a critical step in the purification of marine polyphenolic compounds, where the goal is to separate the desired phenolic compounds from the complex mixture obtained after extraction [41]. The chromatographic techniques are widely utilized for MPC isolation. High-Performance Liquid Chromatography (HPLC) is one of the most used techniques for isolating polyphenols due to its high resolution and ability to handle complex mixtures. It separates compounds based on their interactions with the stationary and mobile phases [42]. Solid-phase extraction (SPE) is a preparative method that concentrates and purifies analytes from a solution by adsorbing them onto a solid phase, followed by elution with a suitable solvent [43]. Flash chromatography is another rapid form of liquid chromatography that uses a pump to pass the solvent through the column, allowing for faster separation and isolation of compounds. The choice of stationary and mobile phases in chromatography is crucial for effectively isolating polyphenolic compounds. The stationary phase is typically a silica-based material, while the mobile phase can be a gradient of solvents varying in polarity. The gradient is optimized based on the polarity of the target compounds to achieve the best separation. The separated compounds are collected in fractions as they elute from the chromatography column. Each fraction is then analyzed to determine the presence of the target polyphenolic compounds. Techniques such as UV-Vis spectroscopy and mass spectrometry are used to analyze the fractions and identify the compounds of interest. For industrial applications, the isolation process needs to be scaled up [36]. This requires careful planning to maintain the separation efficiency at a larger scale. Parameters such as flow rate, column size, and solvent volumes must be optimized for scale-up. After isolation, the purity of the polyphenolic compounds is assessed using analytical techniques such as HPLC, nuclear magnetic resonance (NMR), and mass spectrometry (MS). These techniques provide information on the purity and identity of the isolated compounds, ensuring that they meet the required standards for further use.

Further purification of isolated compounds is essential to obtain high-purity extracts suitable for further analysis and application. The conditions for purification, such as pH, solvent composition, and flow rate, are optimized based on the chemical properties of the target polyphenols. The pH can affect the ionization state of polyphenols, while the solvent composition can influence their solubility and interaction with the purification media [44]. These techniques provide information on the purity and identity of the isolated compounds, ensuring that they meet the required standards for further use. Moreover, recent trends in purification emphasize the use of eco-friendly procedures that minimize the use of harmful solvents and reduce waste. Techniques like aqueous two-phase systems (ATPS) and the use of natural or recyclable adsorbents are being explored to make the purification process more sustainable.

### 2.3. Structural Analysis of Marine Polyphenolic Compounds

**Figure 2:**
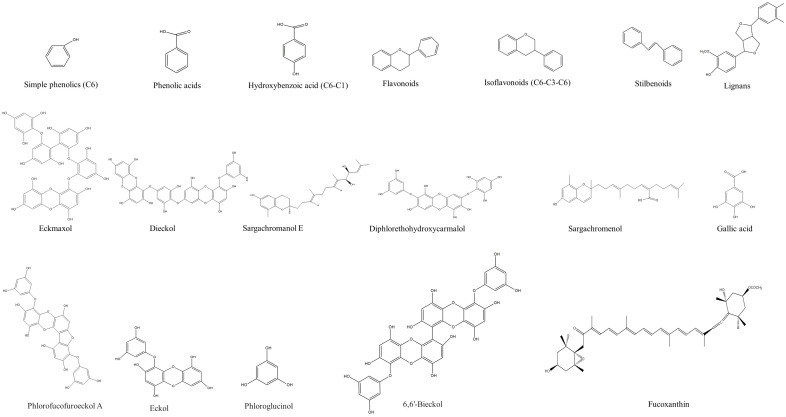
Chemical structures of identified marine polyphenols.

The structure analysis of marine polyphenols is pivotal, not only for elucidating the complex molecular frameworks that confer these compounds their potent bioactivities, such as antioxidant, anti-inflammatory, and cardioprotective effects but also for advancing our understanding of their role in marine ecosystems and their potential applications in nutraceuticals and pharmacology. Recent advances in analytical techniques, including mass spectrometry and NMR spectroscopy, have significantly enhanced our ability to accurately map the complex structures of these compounds, revealing insights into their solubility, stability, and bioavailability. Further, this informs the development of novel marine-derived health products and offers a deeper comprehension of how these natural substances can combat chronic diseases and promote overall health. The novelty and accuracy of structure analysis are crucial as they directly influence the reliability of the biological activity data and the feasibility of translating these findings into clinical and commercial use. The identified chemical structures of MPCs are summarized in Figure 2.

Recent studies have highlighted the importance of total polyphenol content (TPC) in marine and coastal flora, emphasizing the potential of these compounds in food and nutraceutical applications [45]. The therapeutic potential of marine polyphenols extends to their antioxidant, anti-inflammatory, antimicrobial, and antitumor actions, making them promising candidates for mitigating CVDs [45]. The unique chemical structures of MPCs, such as phlorotannin, bromophenols, and phenolic acids, contribute to their diverse biological properties and set them apart from their terrestrial counterparts. The novelty in the content of marine polyphenolics lies in the continuous discovery of new compounds and the advancement of analytical methods that provide deeper insights into their SARs. For instance, the comparison of marine flavonoids with terrestrial polyphenols has revealed distinct structural features that may account for their enhanced bioactivities. This ongoing research is crucial for developing innovative marine-derived health products and for furthering our understanding of how these compounds can be effectively utilized in various industries. The techniques that are used in the current world and their limitations are summarized in Table 3.

## 3. Marine Polyphenolic Compounds against Cardiovascular Diseases

CVDs are a group of disorders affecting the heart and blood vessels. According to the World Health Organization, CVDs are a group of disorders of the heart and blood vessels, including rheumatic and coronary heart diseases, cerebrovascular diseases, and other conditions such as coronary artery disease, pulmonary arterial hypertension, and deep vein thrombosis. These diseases contribute significantly to global mortality, accounting for approximately one-third of all deaths.

**Figure 3:**
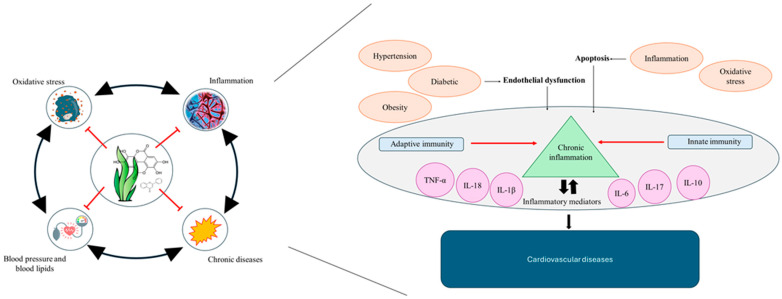
Factors affecting the development of cardiovascular diseases.

Oxidative stress and inflammation are closely linked to the pathogenesis of CVDs, acting as both a cause and consequence of cardiac dysfunction. Further, chronic diseases, diabetes, and hypertension are also critical and can induce oxidative stress and inflammation, further damaging the cardiovascular system. The interplay between these factors creates a vicious cycle where oxidative stress leads to inflammation, which then contributes to the development and progression of diabetes, hypertension, and other chronic diseases, ultimately increasing the risk of cardiovascular events (Figure 3). Table 4 summarizes these activities and their impact on CVDs.

### 3.1. Marine Polyphenolic Compounds with Anti-Hypertensive Activities

MPCs alleviate CVDs in a multifaceted way. Here, some approaches directly regulate CVDs, such as anti-hypertension activities; meanwhile, others provide indirect regulation of CVDs. MPCs exhibit anti-hypertensive activities through various mechanisms, which contribute to their potential in managing high blood pressure.

#### 3.1.1. Angiotensin-Converting Enzyme-Inhibitory Activity

Compounds like eckol, 6,6′-bieckol, and dieckol from *Ecklonia cava* inhibit the angiotensin-converting enzyme (ACE) [46,47]. By blocking ACE, they prevent the conversion of angiotensin I to angiotensin II, a potent vasoconstrictor. This leads to vasodilation and a subsequent reduction in blood pressure. ACE inhibition is a key factor in the anti-hypertensive activity of MPCs. This activity is largely attributed to the structural characteristics of these compounds, which allow them to interact with the ACE and inhibit its function.

The presence of hydrophobic residues, particularly at the C-terminus of the compound, is crucial for ACE inhibition. These residues enhance the binding affinity of the inhibitor to the active site of ACE. Further, the size and shape of the polyphenolic compounds are also vital. Smaller compounds can fit into the active site of ACE more easily, making them more effective inhibitors. The conformation of the compound, whether it is linear or cyclic, can influence its ability to bind to ACE and inhibit its activity. Meanwhile, the charge distribution on the compound’s surface can affect its interaction with ACE. Positively charged areas can attract the negatively charged regions of the enzyme, facilitating a stronger interaction and providing better inhibition.

The ability to form hydrogen bonds with the enzyme’s active site is another characteristic that enhances the ACE-inhibitory activity. Hydroxyl groups in the structure of polyphenols can donate hydrogen bonds, which stabilizes the enzyme-inhibitor complex. Some polyphenolic compounds can chelate metal ions, which are cofactors necessary for the enzymatic activity of ACE. By binding to these metal ions, the compounds can effectively reduce the activity of the enzyme.

These structural characteristics collectively contribute to the ability of marine polyphenolic compounds to inhibit ACE, thereby exerting anti-hypertensive effects. The specific interactions between these compounds and ACE are complex and depend on the precise structure and composition of each compound [46]. Ongoing research continues to explore these interactions to develop more effective anti-hypertensive agents from marine resources.

#### 3.1.2. Modulation of Lipid Metabolism

Fucoxanthin from brown seaweed influences lipid metabolism and improves insulin sensitivity [48]. These effects can contribute to the regulation of blood pressure as they help in reducing the risk factors associated with hypertension. Fucoxanthin has a distinctive molecular structure that includes an allenic bond, a 5,6-monoepoxide, and several hydroxyl groups. These structural features are responsible for its biological activities. The allenic bond present in fucoxanthin is rare in natural compounds and is believed to contribute to its strong antioxidant properties [48]. By combating oxidative stress, fucoxanthin can improve lipid profiles and enhance insulin sensitivity. This functional group 5,6-monoepoxide may play a role in the anti-obesity effects of fucoxanthin by influencing fat metabolism. It is suggested that this structure could affect the expression of genes related to fat metabolism and adipocyte differentiation. The hydroxyl groups in fucoxanthin can engage in hydrogen bonding and other interactions that influence enzyme activity. This can lead to the modulation of enzymes involved in lipid metabolism and glucose regulation. Moreover, Fucoxanthin’s lipophilic nature allows it to incorporate into cell membranes, potentially affecting signaling pathways related to lipid metabolism and insulin sensitivity. When considering the structure characteristics of MPC for the regulation of lipid metabolism, there are five main factors that can be identified.

The initial factor is its polyhydroxyphenol structure. Multiple phenolic structural units characterize marine polyphenols. These structures are critical to their antioxidant properties, which play a role in modulating lipid metabolism. Antioxidants can influence lipid metabolism by reducing oxidative stress, which is known to affect lipid storage and breakdown. The second factor is considered as its lipophilicity. A compound’s lipophilicity degree can determine its ability to interact with lipid membranes and metabolic pathways. Compounds with higher lipophilicity may have better access to lipid-rich areas within cells, thus influencing lipid metabolism directly. The third factor is size and shape, which can affect their ability to interact with enzymes and receptors involved in lipid metabolism. Smaller and more compact molecules may more easily penetrate cell membranes and exert their effects.

The fourth factor is that many polyphenolic compounds occur in conjugated forms with sugar residues. These glycosides can influence the solubility and bioavailability of the compounds, affecting their ability to modulate lipid metabolism. The presence of specific functional groups is considered a final major factor. Certain functional groups, such as hydroxyl groups, can bond with hydrogen and interact with enzymes that regulate lipid metabolism. These interactions can alter the activity of these enzymes and thus influence lipid processing within the body.

#### 3.1.3. Vasorelaxant Properties

Sargachromanol E from *Sargassum siliquastrum* acts as a vasorelaxant. It inhibits calcium channels and reduces oxidative stress, which helps in relaxing the blood vessels and lowering blood pressure [49,50]. Sargachromanol E acts on the vascular smooth muscle cells by inhibiting calcium channels. Normally, calcium ions enter these cells and contribute to the contraction of blood vessels. By blocking these channels, Sargachromanol E reduces the influx of calcium, leading to the relaxation of the muscles in the vessel walls. Furthermore, Sargachromanol E reduces the oxidative stress caused by free radicals, which are unstable molecules that can harm cells and tissues. Sargachromanol E has antioxidant properties that neutralize these free radicals. This reduction in oxidative stress helps protect the blood vessels from damage and inflammation, further contributing to vasorelaxation [51].

The structure of Sargachromanol E includes a chromanol core, which is a 6-hydroxy-chromanol structure. This core is crucial for the compound’s antioxidant activity, as the hydroxyl group positioned at the sixth carbon is highly reactive towards free radicals, aiding in the reduction of oxidative stress. Moreover, Sargachromanol E has various side chains that modify its chromanol core. These modifications can significantly influence the compound’s biological activity. For instance, the presence of preyl groups (such as di-, sesqui-, mono-, and hemiterpenes) attached to the chromanol core can affect the compound’s ability to interact with enzymes and receptors within the cardiovascular system [52]. However, the specific interactions between the structural elements of Sargachromanol E and the biological targets within the cardiovascular system are complex and are a subject of ongoing research.

### 3.2. Marine Polyphenolic Compounds with Antioxidant Activities and Their Potential against Cardiovascular Diseases

Marine flavonoids and phlorotannins, particularly those derived from brown algae, are potent antioxidants with a significant impact on cardiovascular health. Marine flavonoids are a diverse group of polyphenolic compounds found in various marine plants, including algae. They are known for their potent antioxidant properties, which are largely attributed to their chemical structure [23].

The basic structure of flavonoids consists of two aromatic rings (A and B) connected by a three-carbon bridge, forming a third ring. This structure is known as the C6-C3-C6 backbone. The antioxidant activity of flavonoids is influenced by the number and arrangement of hydroxyl groups on these rings. For example, the presence of a double bond between C2 and C3 and an oxo group at C4 on the C ring increases the electron delocalization over the A and B rings, enhancing the radical scavenging ability [53]. As an example, quercetin, a flavonoid present in brown algae *Himanthalia elongate* [54], has a 3-hydroxyflavone backbone and hydroxyl groups at positions 3, 5, 7, 3′, and 4′. The 3′,4′-dihydroxy configuration on the B ring and the 5-OH group are particularly important for its high antioxidant potential [55].

Phlorotannins are unique to brown algae and exhibit strong antioxidant activities due to their polyphenolic structure. Phlorotannins are composed of phloroglucinol units (1,3,5-trihydroxybenzene) linked through various types of bonds, forming complex structures such as fucols, fucophlorethols, and eckols. The number of phloroglucinol units and the type of linkage affect their antioxidant capacity [56]. As an example, eckol, a phlorotannin isolated from brown algae characterized by a dibenzo-1,4-dioxin linkage, has been shown to have significant antioxidant activity. The presence of multiple hydroxyl groups allows for effective radical scavenging. Further, the dimer of eckol, named dieckol, has an even higher number of hydroxyl groups, which enhances its ability to neutralize free radicals and protect against oxidative stress [57]. Table 5 summarizes the MPCs that exhibit an impact on CVDs through their antioxidant activity.

### 3.3. Marine Polyphenolic Compounds with Anti-Inflammatory Activities and Their Potential against Cardiovascular Diseases

Chronic inflammatory conditions are associated with various diseases, including CVDs. Inflammation can lead to endothelial dysfunction, oxidative stress, and vascular damage. Further, these inflammation and oxidative stresses result in narrowed blood vessels that cause hypertension, which is a major risk factor for heart disease. The anti-inflammatory potential of bioactive compounds isolated from marine organisms is well-reported [85]. Therefore, MPCs have a great potential to reduce inflammation and lower blood pressure and other CVDs.

MPCs can inhibit pro-inflammatory signaling pathways, such as nuclear factor-kappa B (NF-κB) and mitogen-activated protein kinases (MAPKs). They suppress the production of pro-inflammatory cytokines such as IL-6, TNF-α, and chemokines. Oxidative stress is closely linked to inflammation [85]. MPCs scavenge free radicals and protect against oxidative damage. By reducing oxidative stress, they indirectly mitigate inflammation. According to previously published studies, MPCs have the potential to significantly suppress the enzymes, including inducible nitric oxide synthase (iNOS) and cyclooxygenase-2 (COX-2). Suppression of these enzymes leads to regulating inflammatory mediators such as nitric oxide (NO) and prostaglandin E-2 (PGE-2) that significantly influence pro-inflammatory cytokine and chemokine production [86].

The aromatic rings and hydroxyl groups of MPCs provide a significant effect on their anti-inflammatory activity. These rings are aromatic and highly reactive, allowing them to interact with cellular components. Further, the hydroxyl groups enhance the antioxidant capacity of polyphenols and increase the potential to combat oxidative stress, a driver of inflammation.

The endothelium, a thin layer of cells lining blood vessels, plays a crucial role in maintaining vascular health. Endothelial dysfunction, often caused by inflammation, contributes to cardiovascular diseases. As mentioned earlier, MPCs alleviate endothelial dysfunction by regulating NO production, oxidative stress, and inflammation-induced damage. Meanwhile, MPCs have the potential to modulate adhesion molecules. Adhesion molecules are proteins expressed on the surface of endothelial cells and immune cells. Their primary role is to facilitate interactions between immune cells and endothelial cells during inflammation [85].

When tissues are inflamed, immune cells such as leukocytes need to migrate from the bloodstream to the affected area. Adhesion molecules play a critical role in this process by acting as “sticky” receptors, allowing leukocytes to attach to the endothelium. Once attached, leukocytes can then migrate through the endothelial layer and reach the inflamed tissue. Several families of adhesion molecules participate in leukocyte–endothelial interactions, such as selectins, integrins, and the immunoglobulin superfamily [85]. MPCs have the potential to downregulate the expression of adhesion molecules on endothelial cells and decrease the availability of binding sites for leukocytes. As a result, fewer leukocytes adhere to the endothelium, leading to reduced inflammation. According to recent studies, marine polyphenols interfere with the interaction between integrins on leukocytes and their ligands (such as ICAM-1) on endothelial cells and disturb this interaction that prevents excessive leukocyte adhesion [87]. Table 5 summarizes the MPCs that exhibit an impact on CVDs through their anti-inflammatory activity.

### 3.4. Metabolic Benefits of Marine Polyphenolic Compounds and Their Impact on Cardiovascular Diseases

MPCs consist of numerous biological activities, such as antioxidant and anti-inflammation, that are beneficial to human health. Apart from that, some MPCs have the potential to directly enhance insulin sensitivity and lipid metabolism that are responsible for CVDs [49,88]. Insulin resistance can alter lipid metabolism, leading to dyslipidemia, which is characterized by high levels of plasma triglycerides, low levels of high-density lipoprotein (HDL), and the presence of small, dense, low-density lipoprotein (LDL) particles [49]. This lipid triad contributes to the development of atherosclerotic plaques in the arteries. Furthermore, aberrant insulin signaling can induce endothelial dysfunction and chronic hyperglycemia that contribute to the progression of atherosclerosis and cardiac metabolic alterations. These changes can lead to cardiomyopathies and coronary heart disease. Lipid metabolism directly affects obesity and CVD risk. The combination of insulin resistance and obesity, especially in individuals with prediabetes or diabetes, significantly increases the risk of developing CVDs [89].

Insulin sensitivity is a critical factor in metabolic health, particularly related to glucose regulation and the prevention of type 2 diabetes. Insulin is produced by the pancreas and plays a vital role in blood glucose metabolism. MPCs exhibit various beneficial bioactivities that contribute to better insulin sensitivity, such as enhanced glucose uptake, activation of adenosine monophosphate-activated protein kinase (AMPK), reduced inflammation, and improved mitochondrial functions [90].

MPCs have the potential to enhance the uptake of glucose by cells by promoting the translocation of glucose transporters to the cell membrane. This results in more glucose entering the cells, which reduces blood glucose levels. MPCs have the potential to activate AMPK, a type of cellular energy sensor that contributes to providing multiple beneficial effects such as increased glucose uptake, enhanced fatty acid oxidation, and suppression of gluconeogenesis [49]. Apart from that, the MPCs regulate insulin sensitivity by decreasing inflammatory conditions and oxidative stresses by controlling pro-inflammatory cytokines such as TNF-α, IL-6, and IL-1β, which interfere with insulin signaling pathways. Healthy mitochondria are crucial for efficient glucose metabolism. Some MPCs have the potential to improve mitochondrial biogenesis and function that enhance mitochondrial functions [49]. Table 5 summarizes the MPCs that exhibit the impact on CVDs through their anti-obesity and insulin sensitivity improvement activity.

## 4. Clinical and Pre-Clinical Evidence

The unique structural diversity of MPCs has been associated with a range of biological activities that are beneficial in the prevention and treatment of CVDs. This specific character of MPCs has gained the attention of scientists and has led to numerous studies. These studies suggest that long-term consumption of polyphenol-rich diets may mitigate oxidative stress and inflammation, which are critical factors in the pathogenesis of CVDs [91,92,93]. Specifically, seaweed polyphenols have been highlighted for their potential role against CVDs [94]. Most studies have identified MPCs as ACE-inhibitors [46,84,95,96]. According to previous studies regarding the ACE inhibitory potential of phlorotannin, Phlorofucofuroeckol A, Flavourzyme digest fraction of E. cava, Methanolic extract of *A. flabelliformis*, inhibited the activity of ACE by 12.74 μM, 0.3 μg/mL, and 13.8 μg/mL IC50, respectively, and the activity is confirmed with positive control Captoprila with 0.05 μg/mL IC50 value [46]. According to Liu et al., most polyphenolic compounds inhibit ACE activity through sequestration of the enzyme metal factor Zn^2+^ ions [97]. Further, the results of these studies have developed a hypothesis that phlorotannins form a complex with surrounding proteins and glycoproteins to inhibit ACE activity. The results from Athukorala and Jeon confirmed that brown seaweed extracts with a higher content of phlorotannins showed remarkable ACE-inhibitory activity. The current study has evaluated water extract from seven seaweed species (*E. cava*, *I. okamurae*, *S. fulvellum*, *S. horneri*, *S. koreanum*, *S. thunbergia*, and *S. lomentaria*), and *E. cava* showed the highest ACE inhibitory activity (36%) [95]. In addition, some MPCs showed vasodilation activity, which is responsible for reducing hypertension. Sargachromenol D, isolated from *Sargassum siliquastrum*, is a vasodilation agonist against endothelin-1 (ET-1)-induced vasoconstriction. The results from this study showed the antagonist effect of Sargachromenol D against ET-1-induced arterial constriction in rabbits at under 9.8 ± 0.6 μM concentration. Further, Sargachromenol D significantly decreased both systolic pressure and diastolic pressure of spontaneous hypertensive rats within 2 h after oral administration of 80 mg/kg, and this effect was maintained for 24 h [98].

The anti-inflammatory and antioxidant activities of MPCs have been confirmed in numerous pre-clinical and clinical studies. Our research team has studied this for years and discovered a plethora of MPCs that exhibit biological activities that directly and indirectly regulate CVDs [13,14,15,99,100,101]. These studies showed that marine seaweed, especially brown seaweeds, contain polyphenolic compounds that have remarkable anti-inflammatory and antioxidant compounds. Apart from that, marine fungi, marine bacteria, and other marine organisms such as microalgae also consist of various polyphenolic secondary metabolites that have anti-inflammatory and antioxidant activity. Xyloketal B represents a polyphenolic compound derived from marine fungi. Zhao et al. (2015) reported that intraperitoneal administration of Xyloketal B (7–28 mg/kg/day) and/or intragastrical administration of 10 mg/kg/day simvastatin as a positive control that Xyloketal B dose-dependently decreased the atherosclerotic plaque area in both the aortic sinus and throughout the aorta in apolipoprotein E-deficient mice (n = 4~6). Further, it significantly reduced vascular oxidative stress levels and improved endothelium integrity and NO-dependent aortic vasorelaxation in atherosclerotic mice. Moreover, Xyloketal B significantly altered the phosphorylation levels of eNOS and Akt in human umbilical vein endothelial cells, increasing phosphorylation at Ser-1177 and inhibiting it at Thr-495, without changing the total expression of eNOS and Akt [102].

Catarino et al. reported the effect of phlorotannins isolated from *Fucus vesiculosus* against metabolic disorders. According to the results, the crude extract and semi-purified phlorotannin fraction showed promising inhibitory effects against α-glucosidase, α-amylase, and pancreatic lipase. A greater inhibitory effect was observed against α-glucosidase compared to the drug acarbose, with an IC50 of 4.5 ± 0.8 μg/mL for the crude extract and 0.82 ± 0.3 μg/mL for the semi-purified fraction versus 206.6 ± 25.1 μg/mL for acarbose. UHPLC-MS analysis identified the presence of fucols, fucophlorethols, fuhalols, and other phlorotannin derivatives, along with possible new compounds like fucofurodiphlorethol, fucofurotriphlorethol, and fucofuropentaphlorethol [60]. Table 5 summarizes the potential of MPCs against CVDs through different therapeutic approaches. According to that, many previous studies have reported that dieckol isolated from brown algal species has remarkable activity against CVDs. Kwak et al. performed a study regarding neurodegenerative diseases by targeting ER stress. In the current study, they utilized fluorescein isothiocyanate (FITC)-labeled dieckol and a rhodamine B-labeled dieckol to determine if they can reach the brain upon systemic administration. According to the results, both compounds successfully crossed the blood–brain barrier (BBB) in rats and localized in the endoplasmic reticulum (ER) of SH-SY5Y and BV-2 brain cell lines and reduced ER stress. Further, the study demonstrates that targeted ER-stress reduction in brain cells can be achieved with fluorone–dieckol conjugates introduced into systemic circulation [103]. The ability of dieckol to penetrate the BBB demonstrates its potential to exert therapeutic effects on target tissues, particularly those impacted by CVDs. Their intracellular localization within the endoplasmic ER of brain cells suggests a capacity for interaction with cellular mechanisms involved in stress responses, which are also related to cardiovascular conditions. Furthermore, the reduction in ER stress by these compounds could be beneficial in mitigating conditions that contribute to CVDs progression, such as atherosclerosis and heart failure. Lastly, their entry into systemic circulation and targeted action point to a possibility for widespread therapeutic benefits, including those affecting the cardiovascular system.

The clinical investigations regarding the MPCs are interesting. Lee et al. confirmed the potential of polyphenol extract isolated from *E. cava* to reduce body fat and oxidative and inflammatory stress in abdominal obesity. The clinical results of this study solidified that MPCs’ consumption led to a significant reduction in body fat from baseline. In subjects with abdominal obesity, MPCs showed a significant reduction in total adipose tissue area, body fat percentage, and fat/lean mass ratio. Moreover, it increases the skeletal muscle index. Moreover, results confirmed that MPCs oxidized low-density lipoprotein levels and showed significant improvement in endogenous antioxidant enzyme activities. Meanwhile, MPCs increased the mRNA expression of genes related to lipid oxidation and decreased the expression of genes related to lipid synthesis. Inflammatory markers were reduced following MPC supplementation [104]. Another recent study has confirmed the beneficial effect of *E. cava* extract on blood glucose and insulin levels in prediabetic patients aged between 18 and 65 years (n = 10). According to the results, a single dose of 600 mg of *E. cava* extracts significantly reduced postprandial blood glucose (PPBG) levels at 90 and 120 min after carbohydrate consumption compared to placebo. The mean prandial blood glucose (PPBG) levels in the seaweed group were 108.1 (±8.9) at 90 min and 101.3 (±8.7) at 120 min versus 122.2 (±16.9) at 90 min and 112.9 (±12.1) at 120 min in the placebo group. Further, no significant differences were observed in the incremental area under the curve (iAUC) of PPBG between the study groups. However, there were no significant differences in postprandial insulin level (PPIL) between the seaweed and placebo groups at any time point, which suggested further research to explore the long-term glycemic modulating effects of MPCs in algal extracts [105]. Feldman et al. investigated the potential of polyphenols against the regulation of dyslipidemia, which is referred to as abnormal lipids in the bloodstream. According to this study, polyphenols have shown health-promoting effects in animal models, particularly in attenuating lipid disorders. Further, clinical trials have demonstrated some benefits of polyphenols on blood lipids and related co-morbidities in humans. However, there is a noted inconsistency in results, which may be due to heterogeneity in studies and challenges in establishing polyphenols’ bioavailability during supplementation [106]. This provides insight into the utilization of MPCs against dyslipidemia by improving lipid metabolism and endothelial function, contributing to reducing the risk of cardiovascular diseases.

HATA et al. reported the clinical effects of *Undaria pinnatifida* on blood pressure. The outcomes of this study revealed that consumption of dried *U. pinnatifida* powder reduced systolic blood pressure (SBP) by 13 mmHg after 4 weeks and by 8 mmHg (*p* < 0.05) after 8 weeks. Further, diastolic blood pressure (DBP) was reduced by 9 mmHg (*p* < 0.01) after 4 weeks and by 8 mmHg (*p* < 0.05) after 8 weeks. Additionally, an 8% decrease in hypercholesterolemia after 4 weeks was observed [107]. This study concludes that *U. pinnatifida* may have beneficial effects as a supplemental treatment for hypertension, which encourages investigation into their composition along with the polyphenolic compounds. However, Murray et al. revealed that the polyphenol-rich fraction of *F. vesiculosus* did not show any blood pressure reduction effect in the Asian population. It also indicated that measuring blood glucose levels alone may not be sufficient to assess diabetes risk in Asian populations, as their plasma insulin responses are consistently elevated [108]. Clinical trials performed with single MPCs are scarce. The scarcity of clinical trials might be due to several factors, including the complexity of conducting studies, the need for extensive funding, and the rigorous regulatory requirements. However, the existing research indicates a significant interest in understanding the role of marine polyphenols in cardiovascular health, which could lead to more comprehensive studies in the future.

## 5. Challenges and Future Directions

Various studies elucidate the value of MPCs against CVDs, as described in the previous sections. However, the scientific community still needs to address certain challenges.

The complexity of the marine ecosystem is one of the significant issues. The marine ecosystem contains many species that can produce polyphenolic compounds. These ecosystems are characterized by their various habitats, such as coral reefs, open oceans, deep-sea vents, and polar waters, each with unique chemical and biological interactions. This complexity poses a challenge for researchers trying to isolate and study specific polyphenolic compounds with potential cardiovascular benefits. The sheer number of species and the interdependence within these ecosystems means that obtaining pure extracts can be difficult. Moreover, environmental factors such as water temperature, salinity, and pressure can affect the composition and concentration of these compounds. Understanding this complexity is crucial for advancing the research on MPCs and their application in treating CVDs. It requires a multidisciplinary approach, combining marine biology, chemistry, and pharmacology, to explore the potential of these compounds.

The complexity of the ecosystem generates a vast structural diversity that challenges establishing SAR. The elucidation of SAR for MPCs is crucial as it provides insights into how specific structural features contribute to their cardioprotective, anti-inflammatory, and antioxidant properties. However, this process is inherently complex and requires sophisticated analytical techniques and computational tools. High-throughput screening methods, combined with advanced spectroscopic techniques such as NMR and MS, are essential for detailed structural characterization. Additionally, computational modeling and molecular docking studies can help predict the biological activity of MPCs based on their structural attributes. As explained in previous studies, specific hydroxyl group arrangements and the degree of polymerization can significantly affect the antioxidant capacity and bioavailability of these compounds [45], and the stereochemistry of MPCs plays a crucial role in their biological interactions and effectiveness [109]. Despite these challenges, recent advances in omics technologies and integrative bioinformatics approaches are facilitating the elucidation of SAR for MPCs. These approaches enable the comprehensive analysis of large datasets, helping to identify key structural features that correlate with biological activity. Continued interdisciplinary research efforts are needed to fully understand the SAR of MPCs, which will be instrumental in harnessing their therapeutic potential for cardiovascular health.

Bioavailability and pharmacokinetics are other critical challenges in the therapeutic potential of MPCs. Despite their promising biological activities, many MPCs face challenges related to poor bioavailability and complex pharmacokinetics, which hinder their effectiveness in vivo. Most MPCs exhibit limited water solubility, which affects their absorption in the gastrointestinal tract [110]. The structural features of polyphenolic compounds are responsible for their low/moderate water solubility [111]. Formulating water-soluble derivatives improves bioavailability in pharmaceutical applications [112]. The solubility of polyphenols mainly depends on their chemical composition and the availability of hydroxyl groups that are attached to the aromatic rings. Therefore, polyphenols are mostly soluble in organic solvents that are less polar than water (ethanol, methanol, and ethyl acetate), and they are commonly used for polyphenol extraction [113]. Glycosylation of polyphenolic compounds is another method to enhance water solubility. Maseda et al. (2023) reported that glycosylation of polyphenolic compounds enhanced their solubility without altering their structure. According to the results of this study, Leloir-type glycosyltransferases catalyzed the regioselective glycosylation of polyphenols. However, they require an expensive and unstable cofactor called UDP-glucose to perform the glycosylation reaction. This approach could revolutionize glycosylation processes for applications in pharmaceuticals, cosmetics, and food industries [114]. As mentioned earlier, solubility is vital for the bioavailability of these polyphenols. The direct and indirect interaction of polyphenols with other compounds, such as proteins or polysaccharides, can affect the solubility of polyphenols. Apart from that, some physiological factors such as pH, transit time, biliary excretion, and intestinal fermentation also affect the polyphenol’s solubility [115]. Further, MPCs can be unstable in the stomach’s acidic environment and can be degraded by digestive enzymes, further reducing their bioavailability [116]. The gastrointestinal microbiota may metabolize MPCs and generate secondary metabolites with different biological activities or that are less bioactive than the parent compounds [117]. Apart from these issues, MPCs may face several pharmacokinetic issues, such as rapid metabolism, which can result in the formation of metabolites with altered or reduced biological activity [118], a short half-life in the bloodstream [119], and limited tissue penetration [120].

The food-related studies also provided evidence of this nature. Ethanol in red wine increases the bioavailability of polyphenols and increases absorption [121]. Further, tartaric acid, which is considered one of the major organic acids in red wine, increases catechin absorption in rats [122]. Meanwhile, the addition of milk to black tea or green tea can inhibit the bioavailability of polyphenols [112]. Jiménez et al. (2023) comprehensively discussed the bioaccessibility and bioavailability of MPCs. The low bioaccessibility of phlorotannins can be explained by their polymeric nature. Further, phlorotannins that have a higher molecular weight tend to bind with proteins and reduce their solubility, challenging the digestive system to break down and absorb them [123]. This was confirmed by our previous study that revealed the antiviral activity of Ishophloroglucin A (IPA) against SARS-CoV-2. The molecular weight of IPA (C96H66O48) is 1986.26 g/mol, and it showed a greater binding affinity with angiotensin-converting enzyme-2, main protease, and papain-like protease enzymes that were responsible for significantly inhibiting viral replication [17].

Nanotechnology-based delivery systems, including nanoparticles, liposomes, or another nanocarrier that can improve solubility, stability, and absorption, can be utilized to enhance the bioavailability of MPCs [124]. Designing a pro-drug is another approach to resolving these limitations. Pro-drugs that undergo enzymatic conversion in the body to release the active MPC can enhance its bioavailability and target specificity [125]. Utilizing bio-enhancers that inhibit efflux transporters or metabolic enzymes [126] and developing controlled-release formulations that can maintain bioavailability in the bloodstream [127] are viable options for enhancing bioavailability.

Safety and toxicity assessment is another vital factor in developing MPC therapeutic agents. Although MPCs have shown promising biological activities, their safe use in humans requires comprehensive evaluation to ensure they do not pose adverse health risks. The lack of standardized protocols and awareness of long-term toxicity is responsible for this issue [128,129]. Chronic toxicity studies are required to assess potential long-term adverse effects, including carcinogenicity, reproductive toxicity, and endocrine disruption. Further, the metabolism of MPCs can produce different or unexpected toxicological profiles compared to the parent compounds. Comprehensive metabolite profiling and toxicity testing of these metabolites are necessary to ensure safety [129]. Apart from that, variations in individual responses due to genetic, environmental, and lifestyle factors can influence the toxicity and safety profile of MPCs. This inter-individual variability must be considered in safety assessments to ensure the compounds are safe for diverse populations. Moreover, interactions with other drugs also have potential limitations that should be overcome [130].

The complexities of conducting clinical trials are another challenge that needs to be addressed. Participant recruitment can be considered a significant issue. Finding enough suitable participants who meet the criteria for a study can be difficult. The participants must represent the population the treatment is intended for and be willing to comply with the study requirements. Current rules and regulations also affect this. Clinical trials are subject to strict regulations to ensure the safety and rights of participants. Navigating these regulations and obtaining the necessary approvals can be lengthy and complex. In addition to these factors, long-term follow-up, cost, and funding are also major issues. Therefore, careful planning, significant resources, and multidisciplinary collaboration are vital for the successful design of further clinical trials. Furthermore, bioavailability can be improved through nanotechnology or other drug delivery systems to ensure that they are absorbed and utilized effectively in the body. Developing better recruitment strategies, such as collaborating with patient advocacy groups and using digital outreach platforms, can help enroll participants who meet the study criteria.

## 6. Conclusions

In conclusion, MPCs hold significant potential against CVDs due to their diverse structures and potent biological activities. The comprehensive analysis presented in this study underscores the potential of MPCs as cardioprotective agents, highlighting their various biological activities, including anti-inflammatory and antioxidant properties that contribute to cardiovascular health. The SAR insights provided herein elucidate how specific structural components of MPCs influence their efficacy, offering valuable guidance for developing targeted therapeutics and nutraceuticals. Despite the encouraging findings, further research is imperative to overcome the challenges associated with the bioavailability, stability, and clinical application of MPCs. Future research should focus on developing more efficient and standardized extraction and purification methods for MPCs to minimize these limitations. This will aid in an efficient approach to SAR studies. Utilizing integrative approaches, such as combining computational modeling, high-throughput screening, and machine learning, can accelerate the elucidation of SAR. Further, these approaches can help identify key structural features responsible for biological activity and predict the efficacy of novel MPCs.

## Figures and Tables

**Table 1:** Comparative overview of marine flavonoid groups.

Group	Structure	Structural Characterization	Common Source	Primary Bioactivity
Flavones	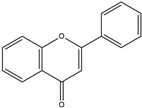	Double bond between C2 and C3, no hydroxyl group at C3	Marine plants	Antioxidant
Flavonols	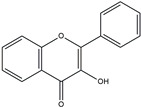	Hydroxyl group at C3	Seaweeds	Anti-inflammatory Cardioprotective
Flavanones	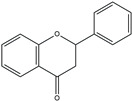	No double bond between C2 and C3 (saturated C chain)	Various marine algae	Antioxidant Anti-inflammatory
Isoflavones	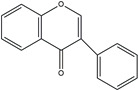	B ring attached at C3 position	Marine fungi and bacteria	Estrogenic Anticancer
Anthocyanidins	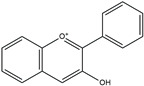	Glycosylated forms of anthocyanins	Some marine plants	Antioxidant
Flavanols	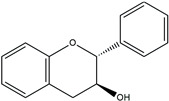	Catechins, often found as part of larger tannin molecules	Green algae, seaweeds	Antioxidant, anti-carcinogenic

**Table 2:** Comparative overview of marine non-flavonoid groups.

Group	Structural Characterization	Common Source	Primary Bioactivity
Phenolic Acids	C6-C1 structure, presence of carboxylic acid group and hydroxyl groups on an aromatic ring	Seaweeds, marine fungi	Antioxidant, radical scavenging
Tannins	High molecular weight, ability to bind and precipitate proteins	Marine plants, algae	Antioxidant
Stilbenes	C6-C2-C6 structure, 1,2-diphenylethylene nucleus	Mangroves, marine sponges	Anti-inflammatory, cardioprotective
Lignans	C6-C3-C6 structure, dimerization of two phenylpropanoid units	Marine algae, seagrasses	Antioxidant, anti-cancer
Phlorotannins	Polymers of phloroglucinol units, varying degrees of polymerization	Brown algae	Antioxidant, anti-inflammatory

**Table 3:** Advanced analytical techniques for marine polyphenolic structure Analysis.

Technique	Purpose	Description	Advantages	Disadvantages
High-Performance Liquid Chromatography (HPLC)	Separation, Identification, Quantification	Utilized for its precision in handling complex samples, essential for polyphenols.	High resolution and sensitivity; suitable for a wide range of polyphenols.	Can be time-consuming; requires derivatization for some compounds.
Gas Chromatography (GC)	Separation	Powerful for analyzing volatile and semi-volatile polyphenolic compounds when coupled with MS (GC-MS).	Good for volatile compounds; high resolution.	Not suitable for high molecular weight or non-volatile compounds.
Nuclear Magnetic Resonance (NMR) Spectroscopy	Structural elucidation	Provides detailed information about the molecular framework and spatial arrangement of atoms.	Non-destructive; provides detailed structural information.	Requires large sample amounts; less sensitive compared to MS.
Mass Spectrometry (MS)	Molecular weight determination	Determines molecular weight and structural features, crucial for structural elucidation.	Highly sensitive and accurate; provides molecular fingerprints.	Complex data analysis; high cost of equipment.
Ultraviolet-visible (UV-Vis) Spectroscopy	Absorbance study	Used to study the absorbance of polyphenols, correlating with structure and concentration.	Simple and quick; good for concentration determination.	Limited structural information; interference from other absorbing species.
Fourier-Transform Infrared (FTIR) Spectroscopy	Functional group analysis	Provides information on the functional groups within the polyphenolic compounds based on their infrared absorption spectra.	Non-destructive; no need for sample preparation.	Lower sensitivity; can be challenging to interpret overlapping peaks.
Capillary Electrophoresis (CE)	Separation, Identification	Separates compounds based on their charge and size under an electric field.	High efficiency and resolution; minimal sample requirement.	Limited to charged or chargeable analytes; lower sensitivity for non-UV absorbing compounds.
Liquid Chromatography-Mass Spectrometry (LC-MS)	Structural elucidation, Quantification	Combines the separation power of LC with the mass analysis capabilities of MS.	Provides detailed structural information; high sensitivity and selectivity.	Requires skilled operation; high cost of maintenance.
High-Resolution Mass Spectrometry (HRMS)	Structural elucidation	Offers accurate mass measurements for precise molecular formula determination.	High accuracy and resolution; useful for complex mixtures.	Expensive; requires expertise for data interpretation.

**Table 4:** Biological activities of marine polyphenolic compounds and their potential impact on cardiovascular diseases.

Primary Bioactivity	Description	Potential Impact on Cardiovascular Diseases	Example	Source
Antioxidant properties	Scavenge free radicals and protect cells from oxidative damage.	Prevents cardiovascular diseases by reducing oxidative stress.	Phlorotannins	*Ecklonia cava* (brown algae)
Anti-inflammatory effects	Modulate inflammatory responses.	Reduces the risk of atherosclerosis and endothelial dysfunction.	Eckol	*Ecklonia stolonifera* (brown algae)
Anti-hypertensive effects	Contribute to blood pressure regulation.	Manages hypertension, a major risk factor for cardiovascular diseases.	Dieckol	*Ecklonia cava* (brown algae)
Anti-hyperlipidemia effects	Regulate lipid metabolism and maintain healthy cholesterol levels.	Reduces the risk of hyperlipidemia-related heart diseases.	Fucoxanthin	*Undaria pinnatifida* (brown algae)
Anti-obesity effects	Aid in weight management and prevent obesity-related complications.	Lowers cardiovascular risks linked to obesity.	Fucosterol	Various brown algae species
Cardioprotective effects	Protect against ischemic heart disease and myocardial infarction.	Provides protection against various cardiac conditions.	Xyloketal B	Marine fungi species
Metabolic benefits	Improve insulin sensitivity and glucose metabolism.	Reduces the risk of type 2 diabetes and metabolic syndrome.	Sargachromanol G	*Sargassum siliquastrum* (brown algae)
Prevention of cardiac fibrosis	Help prevent cardiac fibrosis associated with heart failure.	Aids in preventing conditions that can lead to heart failure.	Saringosterol	*Sargassum muticum* (brown algae)

**Table 5:** The therapeutic potential of marine polyphenolic compounds against cardiovascular diseases.

Disease Type Related to CVDs	Source	MPCs	Assay Type	Activity	Ref.
Diabetes mellitus	*E. cava*	Fucodiphloroethol G Dieckol 6,6′-bieckol 7-phloroeckol Phlorofucofuroeckol-A	In vitro	Inhibition of α-glucosidase IC_50_ values Dieckol: 10.79 μM 7-phloroeckol: 49.49 μM PC—NA Inhibition of α-α amylase Dieckol: 125 μM 7-phloroeckol: 250 μM Other compounds: <500 μM PC—NA	[58]
	*Lessonia trabeculate*	Polyphenol-rich extracts	In vitro	Inhibition of α-glucosidase and lipase activities IC_50_ value <0.25 mg/mL PC—Acarbose—<0.25 mg/mL PC—Orlistat < 0.25 mg/mL	[59]
	*F. vesiculosus*	Crude extract and semi-purified phlorotannins composed by fucols, fucophlorethols, fuhalols, other phlorotannin derivatives	In vitro	Inhibition of α-amylase IC_50_ value 28.8–2.8 μg/mL PC—Acarbose—0.7 μg/mL Inhibition of α-glucosidase IC_50_ value 4.5–0.82 μg/mL PC—Acarbose—206.6 μg/mL Inhibition of pancreatic lipase IC_50_ value 45.9–19.0 μg/mL PC—Orlistat—1.8 μg/mL	[60]
	*Rhodomela confervoides*	3,4-dibromo-5-(2-bromo-3,4-dihydroxy-6-(ethoxymethyl)benzyl)benzene-1,2-diol)	In vitro	Inhibition of PTP1B activity IC_50_ value 0.84 μM Improve insulin sensitivity by activating insulin signaling pathways IC_50_ value 0.1–0.5 μM PC—NA	[61]
	*Rhodomela confervoides*	3-Bromo-4,5-bis(2,3-dibromo-4,5-dihydroxybenzyl)-1,2-benzenediol	In vitro	Inhibition of PTP1B activity IC_50_ value 2 μM PC—NAActivation of insulin signaling and prevent palmitate-induced insulin resistance. IC_50_ value 0.5–2 μM PC—NA	[62]
	*E. stolonifera*	Phlorofucofuroeckol-A	In vitro	Inhibition of AGEs formation IC_50_ value D-ribose-induced insulin glycation: 29.5 μM PC—vanillin—>500 μM D-glucose-induced insulin glycation: 43.55 μM PC—rutin—5.19 μM	[63]
	*Ishige foliacea*	Octaphlorethol A	In vitro	Decreased the death of STZ-treated pancreatic β-cellsDecreased the TBARS and ROSIncreased the activity of antioxidant enzymes IC_50_ value 12.5–50.0 μg/mL PC—NA	[64]
	*E. cava*	6,6-Bieckol, Phloroeckol Dieckol Phlorofucofuroeckol	In vivo	Inhibition of high glucose-induced ROS and cell deathDieckol reduced heart rates, ROS, NO, and lipid peroxidationDieckol reduced the overexpression of iNOS and COX-2 IC_50_ value 10–20 μM PC—NA AM—zebrafish larvae (n = 15)	[65]
	*Ulva prolifera*	Extract rich in flavonoids	In vivo	Diminished the fasting blood glucose and improved oral glucose toleranceHypoglycemic effect by increasing IRS1/PI3K/Akt and suppressing JNK1/2 in liver IC_50_ value 150 mg/kg/day for 4 weeks by gavage PC—NA AM—Kunming male Icr mice (n = 6)	[66]
	*Eisenia bicyclis*	Phloroglucinol, Phloroglucinol tetramer, Eckol Phlorofucofuroeckol A Dieckol 8,8′-bieckol	In vitro	Inhibit β-galactosidase, and β-mannosidase activity PC—NA	[67]
Anti-inflammation/anti-oxidant	*Ecklonia cava*	Eckol	In vitro	Inhibit the expression of TNF-α/IFN-γ-induced inflammatory responses via regulating MAPKs and NF-κB signaling pathway in HaCaT cells PC—NA	[68]
		Dieckol	In vitro and in vivo	Regulate HO-1/Nrf-2 expression in LPS-stimulated macrophages and a periodontitis rat model PC—NA	[45]
		6,6′-Bieckol		Inhibit monocyte-associated vascular dysfunction PC—NA	[69]
		Phlorofucofuroeckol- A	In vitro	Inhibit pro-inflammatory responses in LPS-induced RAW 264.7 macrophages PC—Wortmannin—500 nM	[70]
	*Ecklonia maxima*	Eckmaxol	In vitro	Inhibit pro-inflammatory responses in particulate matter-induced lung macrophages PC—NA	[14]
	*Sargassum horneri*	Sargachromenol	In vitro	Inhibit particulate matter-induced TLR expressions	[13]
	*Ecklonia stolonifera*	Eckol	In vitro	Regulate HO-1/Nrf-2 and inhibit intercellular reactive oxygen species PC—NA	[71]
	*Ecklonia cava*	Dieckol	In vitro	Attenuate cellular lipid peroxidation in keratinocytes exposed to PM10 PC—NA	[72]
	Japanese *Laminariaceae*	1,3,5-trihydroxybenzene Eckol Phlorofucofuroeckol A Dieckol 8,8′-bieckol	In vitro	Inhibition of phospholipid peroxidation in the liposome system PC—NA	[73]
	*Ishige okamurae*	Diphlorethohydroxycarmalol 6,6′-bieckol	In vitro	Inhibit reactive oxygen species in free radical mediate oxidative system PC-α-tocopherol—45.3 μM	[74]
Obesity	*Eisenia bicyclis*	Eckol, Fucofuroeckol A 7-phloroeckol Dioxindehydroeckol Phlorofucofuroeckol A Dieckol	In vitro	Inhibit pancreatic lipase activity IC_50_ value Eckol: 76.6 μM Fucofuroeckol A: 37.2 μM 7-phloroeckol1: 2.7 μM Dioxindehydroeckol: >200 μM Phlorofucofuroeckol A: >200 μM Dieckol: 99.3 μM PC—Orlistat—0.7 μM	[75]
		6,6′-bieckol 6,8′-bieckol 8,8′-bieckol Dieckol Phlorofucofuroeckol-A	In vitro	Inhibit lipid accumulation and adipogenesis by downregulating differentiation of 3T3-L1 adipocytes PC—NA	[76]
	*Ecklonia stolonifera*	Phloroglucinol, Eckol Dieckol Dioxinodehydroeckol Plorofucofuroeckol A	In vitro	Inhibition of lipid accumulation by suppression adipocyte differentiation through inhibiting C/EBPα and PPARγ expression PC—NA	[77]
	*Ecklonia cava*	Dieckol 2,7-phloroglucinol-6 6-bieckol Progallol-Ploroglucinol-6,6-bieckol Phlorofucofuroeckol A	In vitro	Inhibit the leptin resistance PC—NA	[78]
		Dieckol	In vitro and in vivo	Inhibit lipid accumulation and adipogenesis by regulating AMPKα, ERK, and AKT signaling in high-fat diet-fed zebrafish, mice, and 3T3-L1 models PC-Curcumin 2.5 μM AM—mouse (n = 10) and zebrafish larvae (n = 20)	[79]
Hypertension	*Ishige sinicola*	Octaphlorethol A	In vitro	Inhibition of hypertension by inhibiting ACE activity and regulating AMPK and Akt activation IC_50_ value 59 μM	[80]
	*Ishige okamurae*	Diphlorethohydroxycarmalol	In vitro and in vivo	Inhibit hypertension through vasodilation PC—Captopril 10 mg/kg body weight AM: Spontaneously hypertensive rats	[81]
	*Ecklonia stolonifera*	Phloroglucinol Eckstolonol Eckol Phlorofucofuroeckol A Dieckol Triphlorethol-A Fucosterol	In vitro	Inhibit hypertension by inhibiting ACE activity IC_50_ values Phloroglucinol: N.A. Eckstolonol: 410.12 μM Eckol: 70.82 μM Phlorofucofuroeckol A: 12.74 μM Dieckol: 34.25 μM Triphlorethol-A: 700.9 μM Fucosterol: N.A. PC—captopril 1.63–3.26 ng/mL	[82]
	*Ecklonia cava*	Pyrogallol-phloroglucinol-6,6′-bieckol	In vitro and in vivo	Inhibit hypertension by improving blood circulation PC—Ginkgo biloba extract	[83]
		6,6′-Bieckol	In vitro	Inhibit hypertension by regulating ACE activity IC_50_ value 0.42 mM PC—captopril 20 mg/kg body weight AM—Spontaneously hypertensive rats	[47]
		Phloroglucinol Triphlorethol-A Eckol Dieckol Eckstolonol	In vitro	Inhibit hypertension by regulating ACE activity IC_50_ values Phloroglucinol: 2.57 mM Triphlorethol-A: 2.01 mM Eckol: 2.27 mM Dieckol: 1.47 mM Eckstolonol: 2.95 mM PC—NA	[84]

## Data Availability

No new data were created or analyzed in this study. Data sharing is not applicable to this article.
